# Understanding Newcomer Challenges and Opportunities to Accessing Nature and Greenspace in Riverdale, Hamilton, Ontario: A Neighborhood-Centered Photovoice Study

**DOI:** 10.1177/15248399231225927

**Published:** 2024-02-05

**Authors:** Sujane Kandasamy, Matthew Y. Kwan, Parsa K. Memon, Dipika Desai, Russell J. de Souza, Patty Montague, Diana Sherifali, Gita Wahi, Sonia Anand

**Affiliations:** 1 Infant, Child & Youth Health Lab, Department of Child & Youth Studies, Brock University, Ontario, Canada; 2 Chanchlani Research Centre, McMaster University, Hamilton, Ontario, Canada; 3 Department of Kinesiology, McMaster University, Hamilton, Ontario, Canada; 4 Department of Family Medicine, McMaster University, Hamilton, Ontario, Canada; 5 Settlement Worker in Schools (SWIS) Program, YMCA of Hamilton, Burlington & Brantford, Hamilton, Ontario, Canada; 6 Population Health Research Institute, McMaster University, Hamilton, Ontario, Canada; 7 School of Nursing, McMaster University, Hamilt, Hamilton, Ontario, Canadaon, Ontario, Canada; 8 Department of Pediatrics, McMaster University, Hamilton, Ontario, Canada; 9 Department of Medicine, McMaster University, Hamilton, Ontario, Canada

**Keywords:** photovoice, community-based participatory research < health research, health promotion

## Abstract

**Background:**

Access to and engagement with greenspace is related to improved health benefits. We sought to collaborate with community members as partners in research and co-creators in knowledge to better understand which components within a newcomer-dense community help or hinder individual and community efforts to access greenspace and nature-based activities.

**Methods:**

We used photovoice methodology to engage with local residents in focus groups, photowalks, and photo-elicitation interviews. Themes were developed using direct content analysis.

**Results:**

A total of 39 participants (ages 11–70 years; median years in Canada of 3.25 years) were engaged in this program of research. From the analysis, we developed four themes: (a) peace and beauty; (b) memories of home; (c) safety and cleanliness; and (d) welcoming strengthened and new opportunities. Participants associated nature with peace, citing it as “under-rated” but “vital” to the neighborhood. Via photographs and stories, participants also shared a multitude of safety concerns that prevent their access to green/outdoor spaces for healthy active living programs or activities (e.g., woodchip-covered playgrounds, ample amounts of garbage littering the park and school grounds, lack of timely ice removal on sidewalks, limited safe biking paths, and unsafe motor vehicle practices at the crosswalks surrounding local parks).

**Conclusion:**

To translate the key ideas and themes into an informed discussion with policy and decision-makers, we held an in-person exhibition and guided tour where community members, the lead photovoice researcher, and SCORE! principal investigator shared information about each theme in the form of a pseudo-narrative peppered with prepared discussion questions.

## Background

An increasing body of literature demonstrates that spending time in nature can benefit human health in various ways (Fong et al., 2018; James et al., 2016; Kondo et al., 2018; Wendelboe-Nelson et al., 2019), especially during the challenges COVID-19 pandemic (Young et al., 2022). For example, living in greener neighborhoods results in higher levels of physical activity (Browning et al., 2022; Christian et al., 2015; Lovasi et al., 2011) and prevents or mitigates stress, anxiety, and depression in both adults and youths (Berman et al., 2008; Beyer et al., 2014; Maas et al., 2009; Van den Berg et al., 2010; Vella-Brodrick & Gilowska, 2022). Furthermore, greenspace in urban neighborhoods (defined as areas such as gardens, parks, greenways, and other areas with grass, trees, or shrubs) also offers opportunities for neighbors to engage in and promote social cohesion and collective efficacy (Jennings & Bamkole, 2019). Such urban spaces serve as conduits for people to spend time outdoors and interact with nature in ways that may not otherwise be possible. Research also suggests that social cohesion and increased social contacts drive a pathway for which nature and greenspace can also be supportive avenues for public health promotion (Hartig et al., 2014; Oh et al., 2022). Open park designs (Peters et al., 2010), sidewalks (Holtan et al., 2015), shaded areas (Peters et al., 2010), functional playgrounds (Bennet et al., 2012), and organized activities (Plane & Klodawsky, 2013) are factors that help form the connections between urban greenspaces and opportunities for social interaction. Greenspace quality, intended use, and social context impact the level of community member engagement (e.g., environmental stewardship and enthusiasm for volunteering). However, the impositions of modern society centered around technology, high-rise buildings, traffic, and pollution have led to decreased opportunities for community members to interact with nature (Hartig et al., 2014). Indeed growing cities struggle to maintain greenspace, however, research demonstrates a presence of lower canopy coverage in neighborhoods with a higher proportion of lower-income residents (Landry & Chakraborty, 2009) and that higher-income groups have better access to environmental amenities (e.g., public greenspace), compared with lower income groups who lived in places with lower greenspace quality (Kruize, 2007).

This study is nested within the broader “Strengthening Community Roots: Anchoring Newcomers in Wellness and Sustainability” (SCORE!) Research program, whereby the purpose is to co-design and evaluate a suite of healthy active livin interventions for newcomer families living in Riverdale, Hamilton, Ontario (Wahi et al., 2023).

## Objectives

The objective of this study was to collaborate with Riverdale residents by using photovoice methodology to co-create knowledge and understand perspectives and experiences of nature-based programs, activities, and access to greenspace in the neighborhood.

## Methodology and Author Positionality

Underpinned by the lens of critical social theory (Freire’s critical pedagogy), we seek to bring exposure to domination and oppression by positioning participants alongside researchers to collaboratively co-create knowledge through meaningful dialogue (Freire 1972). Originally developed in the 1990s by Wang and Burris (1994, 1997), the overarching objectives of photovoice—a Community-based Participatory Action (CBPA) approach—are to: (1) facilitate participants’ ability to document community strengths and needs; (2) to advance participants’ consciousness of root causes; and (3) to develop data that can be used to advocate for policy change (Wang & Burris, 1997). The photovoice method consists of working with participants from a community who have traditionally lacked influence and engages them in using photography to document the needs and/or strengths of their community. Then, in facilitated group discussions, participants critically discuss the meaning of their photographs, resulting in the enhancement of critical consciousness among participants as it relates to the underlying causes of health issues in their community and potential points of leverage for community-level change. In the end, these points are communicated to policymakers to navigate a conversation for social change (Wang & Burris, 1994; Wang, 1999). Because visuals can operate as signifiers of culture, emphasizing values and expectations of individuals, communities, and society, what is selected to be photographed, when and how, is guided by individual and community values. Likewise, interpreting images is a subjective process and informed by personal meaning (Dicks et al., 2006; Edwards, 2002; Orellana, 1999). It is for this latter reason—seeking to understand the meaning that participants bring to images—that we selected this methodology to explore our research question. Images shift the power dynamics and allow researchers and participants to be counterparts in the exploration of societal challenges, catalyzing opportunities for new and deeper understandings (Harper, 2002; Liebenberg, 2009).

One of the main purposes of using a CBPA approach such as photovoice is to re-establish the research ecosystem between participants and researchers using approaches that minimize power differentials and seek to equalize roles (Farquhar et al., 2014; Israel et al., 2019; Stoeker, 2009). Participants essentially become co-researchers in the process, contributing and helping to guide the course of data collection and analysis. It leads to new innovations in research findings (e.g., the development of themes that researchers may not have previously been exposed to or understandings that researchers have traditionally undervalued, underappreciated, or have been sensitized to).

This study was led by a postdoctoral researcher who self-identifies as a Tamil-speaking member of the South Asian diaspora living in Canada (SK). She is a woman of color, parent, long-time advocate for environmental stewardship, and has doctoral-level training in mixed-methods and qualitative research. Members of the team include researchers and community members who are passionate about supporting and improving the lives of those living in Riverdale through research, scholarship, and community-based social infrastructure. They bring unique experiences and expertise in school-based community engagement (PKM and MYK), pediatric medicine (GW), epidemiology (SSA and RJD), adult cardiovascular disease (SSA), physical activity (MYK), youth mental health/wellness (GW and MYK), community engagement (SK, GW, and SSA), and co-design methodology for chronic disease prevention/management (GW, DS, and SSA). Finally, it is a vital component of the SCORE! Research program to use research methodologies that allow for genuine community engagement and active participation among those who live in the Riverdale community. We approach this work with the understanding that community members are co-researchers across all stages of the research process, and through the use of CBPA, we seek to redistribute power (Farquhar et al., 2014; Israel et al., 2019; Stoeker, 2009) to meaningfully foster self-efficacy, trust, and mutual commitment (Israel et al., 2019).

This study used a combination of participant-centered methods to collect and analyze data. These methods included focus groups and a group activity (photography mission).

**Figure 1:**
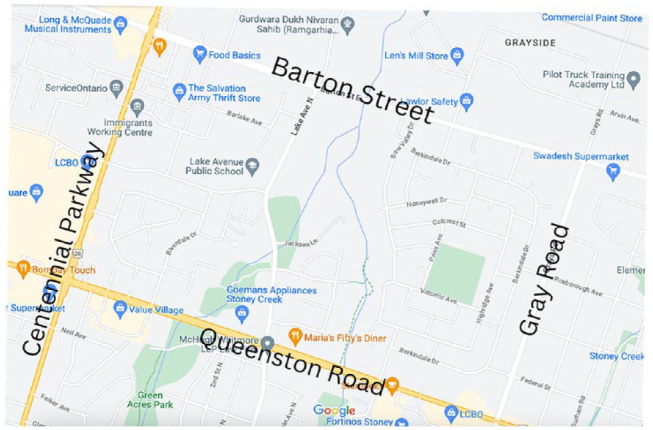
Map of Riverdale, an “Arrival Neighbourhood” in Hamilton, Ontario (Geographic Site of This Research Project)

Taking place in an inner-city neighborhood located in Hamilton, Ontario, Riverdale, consists of East and West components and is bordered by Barton Street, Grays Road, Queenston Road, and Centennial Parkway (Figure 1). Colloquially, it is considered Hamilton’s “arrival city,” where many newcomers (those who have arrived in the past 5 years) settle when they first start their lives in Canada (25% of immigrants have arrived to Canada within the last two decades) (Statistics Canada, 2021). In 2014, Hamilton also declared itself a “sanctuary city” for undocumented immigrants—an idea where the city takes deliberate steps to ensure that municipal services (e.g., community housing, health care, social assistance, education, etc.)are fully accessible to all residents, regardless of immigration status (Nursall, 2014). Notably, 50% of Riverdale residents identify as a visible minority, and the community is ethnically diverse, largely composed of young families from South Asia, Southeast Asia, and the Middle East (Census Mapper, 2021). Over 25% of residents identify as low-income (Statistics Canada, 2021), despite that many have arrived from their homeland with advanced degrees in medicine and engineering.

In collaboration, SK and PMK engaged with diverse community partners who work closely with newcomers living in the Riverdale neighborhood to promote research activities, recruit participants, select physical space to host in-person research activities, and synergize on knowledge mobilization outputs (e.g., exhibition, social media promotion, etc.). We also carefully reviewed enrollment after each community photowalk to better tailor future research activities to include “missing voices.” Community partners served a key role in that tailoring process. For example, we aimed to ensure the inclusion of diverse genders, ages, and ethnic backgrounds, which was led by different community partners and in a staged approach to ensure meaningful capture of diverse community voices.

This study received ethics approval from the Hamilton Integrated Research Ethics Board on March 14, 2022 (Project ID #: 14272).

In terms of study processes (data collection and analysis), after the study was explained and discussed in detail with all interested participants, written informed consent was obtained prior to proceeding with the interviews and photowalks. The majority of participants used their own smartphones to take photos. If they did not have one, disposable cameras were available on-site for them to use during the photowalk. Data collection included the following key steps:

1. Initial focus group: partnering organizations provided in-kind space (which included a private technology-enabled room with tables and chairs). We used this space to complete initial questions around perceptions of nature and access to greenspace in the Riverdale neighborhood. Focus groups were conducted as a combination of English and native languages (e.g., Arabic and Pashtu). The lead researcher and a translator worked together to ask questions and follow-up prompts with two-way translations happening as needed. All focus groups were audio-recorded and only the English translations were transcribed.
2. Photowalk: from the discussions that occurred during the initial focus group, we co-selected key community landmarks to plan out the group photowalk. We proceeded together on the pre-selected route, walking slowly to allow participants to stop and take photos and/or share their narratives. Additional landmarks were visited in real-time as per request by participants. Participants were given the research task of taking photos of their perceptions of nature/greenspace (broadly and locally) and barriers and facilitators to accessing nature/greenspace for healthy active living.
3. Photo-elicitation focus group: after the completion of the photowalk, we returned to the community space to engage in the series of final focus group questions where participants were asked to electronically share a selection of the photos they took or had previously taken and the story behind it. Questions were designed using the SHOWED method (Wang & Burris, 1997) and can be found in Appendix A. This focus group was also conducted as a combination between English and native languages (e.g., Arabic and Pashtu). The lead researcher (SK) and a translator worked together to ask questions and follow-up prompts with two-way translations, in real-time, happening as needed. In addition to the photos participants took during the photowalk, participants were also invited to share photos they had taken previously if they related to the research question. In some cases, participants had photos showcasing their perspectives of nature and greenspace (the first part of the photography mission) that may have been taken outside of Riverdale (i.e., in their homeland as many had immigrated within the last year). All focus groups were audio-recorded and only the English translations were transcribed.

Data were analyzed using an iterative and inductive process centered on direct content analysis. After all focus groups were transcribed verbatim, transcripts were analyzed manually through the principles of open, focused, and thematic coding (Braun & Clarke, 2006; Pope et al., 2000). Photographs and associated descriptions (or narratives) that reflected the “photography mission” were shared by participants during the photo-elicitation focus group. Codification of photographs (i.e., labeling them based on key factors such as barriers facilitators, etc.) was done alongside participants. All focus group data were analyzed after the completion of the workshop and co-developed by the investigative team, which included a key community beneficiary. We also conducted a “member check” of the data at community events and community advisory board meetings, which included local newcomer voices.

## Results

Recruitment and data collection for this study occurred between July and December of 2022. During this time, 39 participants were enrolled and completed the study (pre-photowalk focus group, photowalk [photography mission], and photo-elicitation focus group). All data were collected in-person and face-to-face. The median age was 19 years (11*–*70), with 28% identifying as a woman; 21% identifying as a man; 28% identifying as a girl; and 23% identifying as a boy; zero participants identified as gender-diverse. Participants spent a median of 3.25 (0.125–18) years in Canada with 72% identifying with Middle Eastern ethnicity, 18% identifying as South Asian, and 10% identifying as North or East African. In terms of immigration status, most identified as permanent residents (67%), with some being citizens (28%) or temporary status (5%). The majority of participants were students (56%), with some being employed full or part-time (23%) or unemployed (21%) (see Table 1).

**Table 1:** Demographic Details of Photovoice Participants

Age (*N* = 39)	Immigration status (*N* = 39)
Median: 19 (11–70)	Permanent resident: 26
Gender (*N* = 39)	Canadian citizen: 11
Women: 11	Temporary status: 2
Men: 8	Employment status (*N* = 39)
Girl: 11 Boy: 9	Employed (full-time or part-time): 9
	Unemployed: 8
Gender-diverse: 0	Student: 22
No. of years in Canada (*N* = 39)	Living situation (*N* = 39)
	Rental properties: 39
Median: 3.25 (0.125–18)	
Self-reported ethnicity (*N* = 39)	Apartment: 22
Middle Eastern: 28	Townhouse: 14
South Asian: 7	Detached house: 3
African (North and East): 4	

In total, 161 photographs illustrating the individual and community-level barriers and facilitators to accessing greenspace and nature were shared by the 39 participants. Through these photos, four themes developed from the complementary and interconnected visual and narrative data uncovered intrapersonal, socio-cultural, and environmental factors related to experiences with greenspace and nature: (a) peace and beauty; (b) memories of home; (c) safety and cleanliness; and (d) strengthening existing opportunities and welcoming new ones. After classification, 112 photographs related to the peace and beauty of nature; 7 photographs to memories of the homeland; 18 photographs to safety and cleanliness; and 24 photographs to strengthening existing opportunities and welcoming new ones.

**Figure 2:**
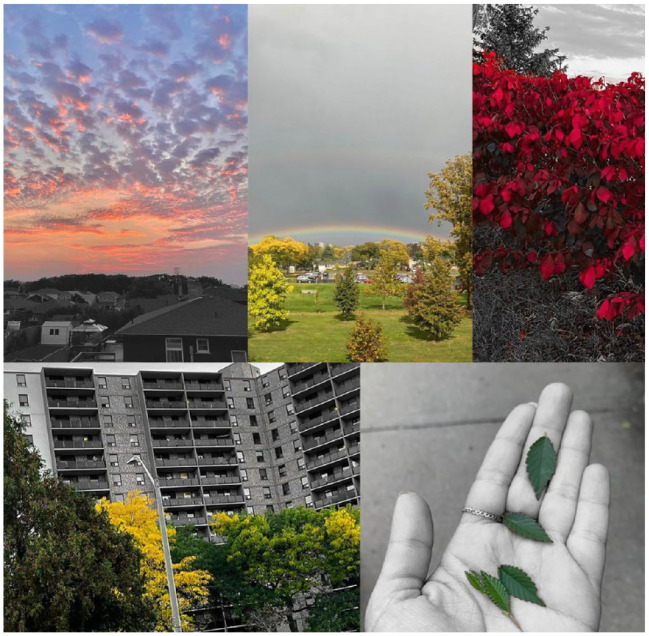
Participant Photographs Relating to Nature in Riverdale Being Associated With Peace and Beauty *Note.* (From left to right, top to bottom): The first image describes the beauty of the sunset; the second illustrates the beauty of a rainbow after the rain; the third is of red-colored leaves; the fourth is of the changing leaf colors during autumn; and the fifth is of the beauty of leaves and the representation of how *“trees connect us, every root”* (Participant, Photowalk #2).

Peace and beauty: Participants described nature and greenspace as encompassing characteristics of peace and beauty, bringing humans a deep sense of pleasure. A select number of photos representing the 112 total photographs related to this first theme of peace and beauty are shown in Figure 2. These images described the attraction of differently shaped leaves, the sky and clouds (especially how the sky changed color throughout the day—moving from the bright warm colors of the sunrise to the cool colors during the day, and then back to a warm color palette during sunset), changing seasons (e.g., witnessing the leaves changing color during the autumn months), and rare but spectacular sightings such as rainbows. They enjoyed spending time in nature to experience these types of sightings. They also articulated how vital but undervalued nature in the community was to them as residents, particularly with the progression of local industrialization, urban renovations, and construction. Participants also indicated that there are very few areas in the community that are “purely natural,” as the local parks, tree-lined streets, and fields are often constructed by humans. Participants also made the connection between natural spaces and health/well-being. For example, the following participant says,

Most parts have been touched and they have been occupied and they have been shaped by the human and their activities. So, we see some examples, but there are not enough . . . We need further nature. We need to pay more attention, and the government, the municipalities have to provide further natural spaces for the people, if there is more natural spaces, we’ll live healthier, and we live longer (Participant, Photowalk #4).

Participants also wanted to see more greenery and gardens—for example, one mother said that would help *“better her mental health”* (Participant, Photowalk #1).

**Figure 3:**
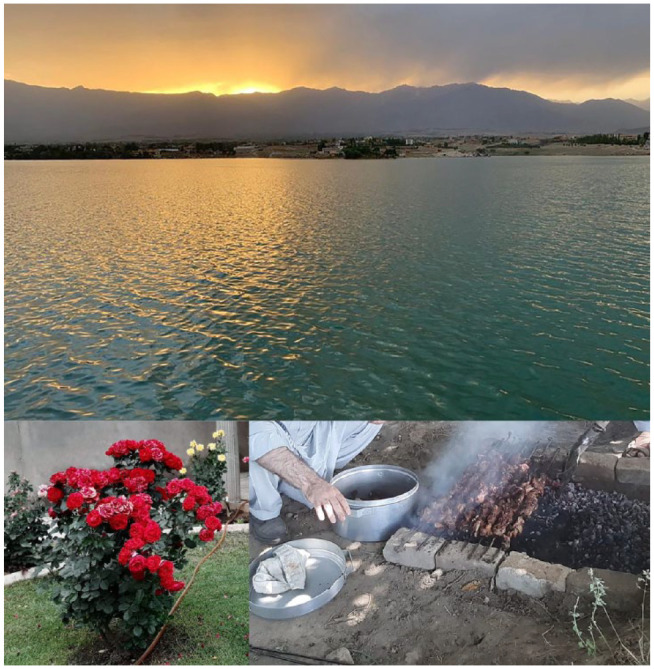
Participants Shared Photos of Nature From Their Homeland and How the Memories Stemming From These Experiences Encouraged and Inspired Them to Access Similar Opportunities in Riverdale and the Broader Hamilton Community *Note.* The first image relates to the Kabul mountains and lakes; the second relates to large-petaled roses grown in residential backyards; and the third relates to social cooking and food preparation activities that happen outdoors. Participants connected these memories to their past (living in another country—most were living in the Middle East) and present/future (living in Riverdale).

Memories of home: A total of seven photographs were submitted related to the theme of memories of one’s homeland, and a selection of these is included in Figure 3. Participants (especially individuals who had more recently immigrated to Canada) spoke at length about how their memories of landscapes and nature from their homelands often guided the types of places and spaces they were attracted to or more likely to visit after moving to Canada. For example, memories of the Kabul mountains and lakes inspired them to explore analogous landscapes in the Hamilton waterfront area. Furthermore, memories of outdoor cooking and meal sharing with family members and friends enthused them to plan similar activities in local Hamilton parks and waterfall trails. For example, one participant says,

One of the photos that I sent, it was from Kabul. There is a lake in the west side of Kabul called Qargha Lake. The reason I sent that picture, I have a really good memory from that place. Every time that people are getting tired, and people are going to there and then drinking tea, enjoying, and riding a horse, and then just having fun. It is connected to part of my quite long journey that I started of coming from Kabul to here. So, it is connected, those pictures, memories with me and with my future (Participant, Photowalk #4).

**Figure 4:**
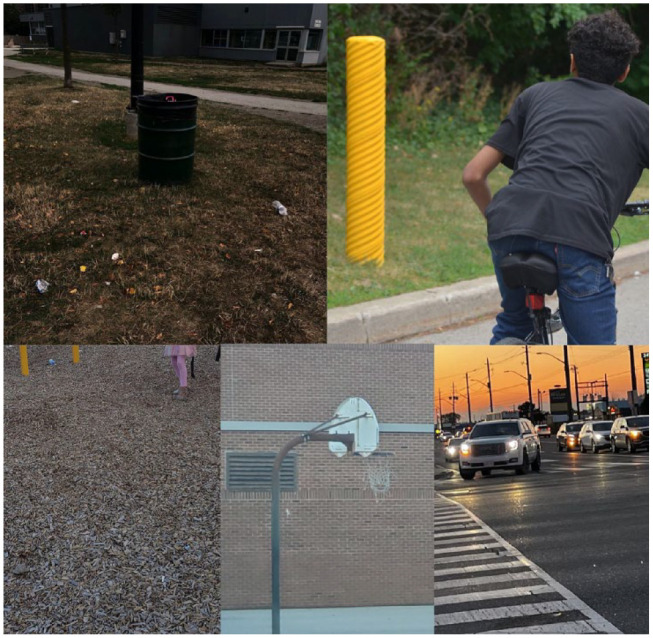
Photographs Representing Safety and Cleanliness Concerns *Note.* The first image depicts the single garbage can in Lake Avenue Park (with plastic and glass waste littered on the grass outside of the garbage can). The second image depicts the need for safer bike lanes and paths. They also discussed bikes being frequently stolen: *“I swear, if you have a bike, the next day you won’t see it. You’re out of luck. I swear”* (Participant, Photowalk #2). The third image depicts the woodchip-covered playground. One participant says, *“woodchips are dangerous for kids and there should be another option. [There’s] a park in Burlington that has a foam ground. Woodchips also gets the kids dirty. I think [woodchips] are the biggest thing”* (Participant, Photowalk #1). The fourth image represents the damaged basketball nets and the final image represents the ample amounts of cars and speeding that occurs on the major streets surrounding the Riverdale neighborhood.

Safety and cleanliness (Figure 4): Many participants agree that although there are compelling components of the community’s natural environment, there are also some concerns and challenges (18 photographs total). These relate particularly to safety and cleanliness. Experiences of nature cross all senses—the smell of the rain, the touch of leaves, the sight of waterfalls, but also disruptions to these experiences, such as the sound of traffic—of cars and large trucks traveling at high speeds through the neighborhood. They all feel the crosswalks can be unsafe at times, including the ones around the two local parks. They are perceived to be even more unsafe in the later evening hours when streetlights are burnt out or not working. They also say that ice and snow are not quickly cleared off sidewalks, making winter outdoor walking more challenging. Many reported winter slips and falls while walking outdoors, describing winter as a period where they prefer to stay indoors wrapped up in warm blankets. During the private guided tour, some participants also described the isolating aspects of the winter months, especially for older adults and seniors.

The Lake Avenue Park is a vital hub for many families. It is here that you can witness gatherings, connections, and outdoor play happen during the summer months (including well into the evening hours). Children and youth of all ages can be found using the swings, fields, and basketball nets for free play. However, the mothers and many youths express concerns over the woodchip-covered grounds—they feel it is unsafe for children, especially the younger ones. They also worry about the garbage and litter—the plastic and glass waste that can be seen sprinkled on the grass and fields. Many reflected on the single garbage can in the whole park and how the children and youth barely make use of it. They compare the services with other parts of the city where there are more consistent clean-up efforts and more greenspace. Many of the male youths report that some of the equipment, particularly the basketball nets, could benefit from upgrades. Nets have been reported to be broken, bent, ripped, and have even fallen over causing injuries.

**Figure 5:**
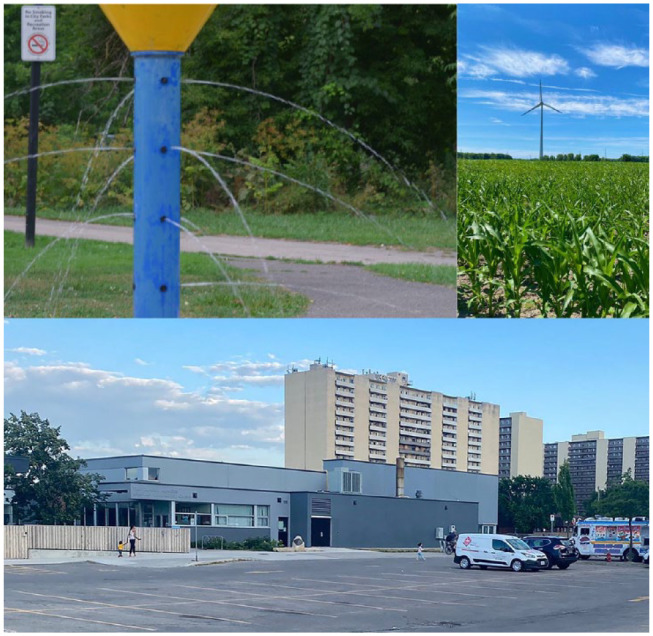
The First Image Represents the Hope for More Splashpads Within Walking Distance to Riverdale Residents *Note.* Some mothers said, “I drive a distance to get my kids to the water park and they really enjoy that. I would like to see more splash pads here” (Participant, Photowalk #1). The second image depicts the love for visiting farms (and participating in activities such as apple picking) outside of the Riverdale community. Finally, the third image depicts the Dominic Agostino Riverdale Community Center, a community hub. People enjoy gathering here for structured activities, free play, and pastimes such as enjoying a nice cold ice cream cone on a hot summer day.

Strengthening existing opportunities and welcoming new ones (Figure 5): A total of 24 photographs were submitted related to this theme. Many participants wanted to see strengthened local opportunities and were at the same time, open to welcoming accessing places and spaces outside of Riverdale. Strengthened local opportunities they wished to see more of are safer biking trails and more splash pads that are located within the community. They also appreciate the diverse drop-in programs at the Dominic Agostino Community Center and the ones offered through the YMCA; however, they wished to see more of these programs offered, especially since many were canceled during and after the COVID-19 pandemic. Many participants, especially the female youths want to access opportunities outside of the community, particularly referencing apple picking and farm visits. For example, one participant said the following,

I really like going there, because it’s like really nice. It’s really quiet. It’s really peaceful, away from the like the city. I think it’s really peaceful there. And the greenery, and everything’s just so nice. That’s why everything’s natural. Like, cows’ milk, and honey and everything (Participant, Photowalk #3).

Transportation was described as a challenge for many, and must be considered an important barrier to accessing services, programs, activities, and open free greenspace (trails, waterfalls, etc.) outside of the neighborhood borders.

In early March of 2023, we held an in-person photovoice exhibition. We threaded together a narrative showcasing a unified perspective of all the corresponding stories. It was an open event for all community members to participate in “member-checking” activities. In particular, 5 newcomer Riverdale families with children who live with disabilities and 26 local decision-makers were invited to attend a private tour of the exhibition to engage in solutions-centered dialogue. The objective of this effort was to facilitate participant civic engagement while strengthening the conversation around collaborative efforts to improve greenspace and access to more healthy active opportunities.

We developed a digital story to capture the exhibition, which can be watched here: https://drive.google.com/file/d/13feDmnmHh0khXttzO7febd8rwkGz5Sl2/view?usp=sharing

## Discussion

This study represents the first study focused on characterizing the environmental links to nature, safety, and well-being in a newcomer-dense neighborhood. We demonstrate that Riverdale residents associate nature with “peace and beauty,” citing it as “under-rated” but “vital” to the neighborhood. Via photographs and stories, we showcase a multitude of safety concerns that prevent community access to green/outdoor spaces for healthy active living programs or activities. These safety concerns are currently being communicated to policy/decision-makers to collaborate on solutions and action.

Exploring this research question using a participatory research methodology such as photovoice centers principles of community engagement, empowerment, and social change. This approach enables community members to visually capture and document their daily experiences, issues, and concerns, and then to use these images to generate dialogue, reflection, and action on social issues that directly affect their lives. Photovoice is increasingly being used in various fields, such as public health, urban planning, and social policy, to enhance community engagement and civic change (e.g., Damian et al., 2022; Gómez-Varo et al., 2023; Macdonald et al., 2022). One of the key benefits of photovoice is that it provides a platform for marginalized and underrepresented communities to amplify their voices, enabling the mobilization of issues that may otherwise go unnoticed or ignored. By using photography to capture experiences, community members are empowered to take an active role in identifying and addressing the social determinants of health, such as poverty, environmental degradation, and discrimination. By facilitating community members to share their images and stories with others, photovoice can create a space for dialogue and exchange of ideas, experiences, and perspectives. This process can help to build trust, understanding, and solidarity among community members, and can lead to the development of collective actions to address common issues and concerns. In addition, photovoice can facilitate the engagement of policymakers, professionals, and other interested parties in the community, who can learn from and respond to the insights and experiences of community members. Photovoice can provide rich and nuanced data on the social, cultural, and environmental factors that influence health and well-being which can be used to inform policy and program development, advocacy, and community-based interventions that promote equity and social justice (e.g., Haque et al., 2018; Yang et al., 2020; Goodman et al., 2019; Tobin et al., 2023).

A recent systematic review reports that interactive sensory connections (e.g., observing greenspace, olfactory experiences with weather, etc.) fostered new memories that facilitate adaptation and attachment to new natural environments (Charles-Rodriguez et al., 2023). The same review reports that social interactions and reconnecting with pre-migration experiences promote cultural continuation and a sense of belonging and well-being (Charles-Rodriguez et al., 2023). This is of importance because pre-migration experiences play a large role in seeking opportunities for re-connection—which was also a shared perspective among Riverdale participants in this study.

Canadian-based studies have also demonstrated that activities in the natural environment serve as a protective factor in the health and well-being of immigrant children and families, mainly through emotional and physical nourishment in the face of adversity (Hordyk et al., 2015). Further research has demonstrated that immigrants’ participation in nature-based activities can foster integration, belonging, place attachment, well-being, and physical activity (Charles-Rodriguez et al., 2023) and promote immigrants’ well-being and function as protective factors against acculturative stress (Stodolska et al., 2017). Although participation in natural environments can offer restoration, leisure, stress reduction, and feelings of joy, safety, and peace, it can also prompt negative feelings such as fear, isolation, and/or insecurity. Further research is required to better understand the pathways between participation in natural environments and immigrants’ well-being.

## Implications for Practice and/or Policy and Research

Through consideration of key community partner perspectives and working collaboratively with municipal partners in a policy roundtable, a strength of this research is that it develops a pathway to curate, communicate, and co-create solutions to key areas of concern discussed by participants. To foster dialogue with city leaders and local champions, we have collated key action points (which can be found in Table 2) to share, brainstorm, and seek commitment for actionable change. Although the specific action points may necessarily be transferrable to other communities, the key messages around building safer spaces through strengthening local opportunities for newcomer families to engage with greenspace are transferrable. These include clean parks (with ample garbage and recycling bins) and enough space for community children and youth to play, safer motor traffic surrounding schools and parks, and upkeep park equipment (e.g., basketball nets). Furthermore, we build a case for using photovoice methodology as an avenue to include local residents in policy-change initiatives. This methodology allows participants to effectively and clearly share concerns, challenges, and community strengths using their own voices and imagery. These data sources can be shared directly with policy/decision-makers so the commentary provided by those who are often silenced in policy-building conversations can be heard loud, clear, and in their own words.

**Table 2:** Lower and Greater Resource Solutions

Lower-resource solutions	Greater-resource solutions
Garbage and litter covering the major source of greenspace in the community can be minimized by having more garbage/recycling cans in the Lake Avenue Park, introducing clean-up programs, and supplemental school-based workshops focused on improved waste management (refuse, reduce, reuse, recycle)	Building a larger inclusively-designed playground with splashpads
Planting of more trees and gardens with the support of local partner organizations	Construction of safe bike paths and communal bike storage facilities
Timely clearing of ice and snow from public spaces (including sidewalks)	Implementation of more robust pedestrian-friendly practices around the local parks, schools, and gathering spaces

## Conclusion

This study demonstrates that among newcomers to Canada, Riverdale community residents associate nature with “peace and beauty,” citing it as “under-rated” but “vital” to their neighborhood. Via photographs and stories, they shared a multitude of safety concerns that prevent their access to green/outdoor spaces for healthy active living programs or activities including garbage littering the park and school ground and unsafe motor vehicle practices at the crosswalks surrounding local parks. We are now engaging policymakers and local decision-makers to continue to curate, communicate, and co-design on solutions to improve access to greenspace and nature-based opportunities for healthy active living.

## Appendix A

Interview 1 (before the photographs are taken):

1. When you think about nature, what comes to mind?
2. What is the nature like in your neighborhood?
  How often do you go to the nature areas that you mention?
  What do you like about the nature in your neighborhood?
  What don’t you like about the nature in your neighborhood?

3. What would encourage you to spend more time in the nature in your neighborhood?
4. What other nature areas would you like to see or visit?
  - ideal situation of what is available in your neighborhood in terms of greenspace

5. Any other comments about nature or nature in your neighborhood?
  - send notifications once a week to remind participants
  - explain the different definitions right in the app (and just include barrier and facilitator)
  - user-friendly app

Interview 2 (after the photographs are taken, based on SHOWED criteria):

1. Why did you take this photo?
2. What does it mean to you? Why?
3. What is really happening here? Why does this barrier, facilitator, or situation exist?
4. How does it relate to our lives?
5. What can we do about it?
